# Process and Outcome Evaluation of Integrating Primary Eye Care into Primary Healthcare: A Quasi-Experimental Study in Rural China

**DOI:** 10.5334/ijic.8972

**Published:** 2026-02-13

**Authors:** Xiaodong Dong, Ziyin Zhao, Jin Xu, Xiaochen Ma

**Affiliations:** 1 School of Public Health, Peking University, Beijing, China; 2 China Center for Health Development Studies, Peking University, Beijing, China; 3 National Health Commission Key Laboratory of Health System Reform and Governance, Peking University, Beijing, China

**Keywords:** primary eye care, primary healthcare, vertically integrated health system, process and outcome evaluation, context, quasi-experimental study

## Abstract

**Introduction:**

Visual impairment is a critical issue in low- and middle-income countries (LMICs), where unmet eye care needs are significant. The WHO’s 2019 World Report on Vision recommends integrating primary eye care (PEC) into primary healthcare (PHC). This study evaluates the integration of PEC into PHC within County-wide Tight Medical Alliances (CTMAs) in rural China.

**Methods:**

From 2021 to 2023, we implemented the *Eye CARE Model*, focusing on Capacity building, Awareness raising, and Referral system Establishment (CARE). We used the Medical Research Council (MRC) process evaluation framework and a quasi-experimental difference-in-differences (DID) method to assess the impact on eye care utilization in three pilot counties in Yunnan Province.

**Results:**

The *Eye CARE Model* increased primary and secondary eye care visits by 76 and 52 per 10,000 people, respectively. It also improved community-based eye health education and established a three-tiered referral system. The intervention had high fidelity and reach, supported by local government and CTMAs, though challenges in sustaining incentives and integrating PEC indicators into health systems were noted.

**Conclusions:**

The Eye CARE Model successfully increased eye care utilization and demonstrated the feasibility of integrating PEC into PHC in rural China, emphasizing the importance of contextual factors in LMICs.

## Introduction

Visual impairment (VI) is a critical public health issue affecting a substantial number of people globally, nearly 90% of them live in low- and middle-income countries (LMICs) [1,2]. However, the unmet needs in eye care in high in LMICs, partly due to lack of eye care resources and the maldistribution of the facilities and workforce [2,3,4]. In order to address the unmet needs in eye care services, the WHO *World Report on Vision* 2019 proposed the concept of Primary Eye Care (PEC) which moves the service delivery to primary-care- and community-based, integration with other primary healthcare (PHC) services [2]. PEC typically includes health promotion activities, diagnosis and treatment of simple eye conditions, detection and referral of emergencies, cataracts, and other causes of visual impairment, as well as rehabilitation services, aligning inherently with the functional mandate of PHC [1,2].

Review of recent evaluations of PEC interventions suggests that translating these strategies into routine practice faced challenges [5,6,7]. One of commonly reported barriers was the complex process of health system strengthening therefore simply enhancing service delivery capabilities might not achieve intended consequence [7,8,9,10]. For example, PEC training, task shifting, and task sharing designed to improve care quality and increased access to eye care services, with impacts remained constrained by their failure to embed within existing healthcare systems or lack sustainable incentive mechanisms that address both supply and demand sides [11,12,13]. Moreover, despite efforts to enhance community awareness and trust, long-term sustainability remains uncertain [13,14,15]. Addressing the multifaceted challenges of integrating PEC into PHC in LMICs necessitates comprehensive, multi-component intervention strategies that generate cohesive system-wide change.

To address these limitations, we designed a system-integrated multicomponent intervention for integrating PEC into PHC in rural China entitled the *Eye CARE Model*, which encompassed Capacity building, Awareness raising, and Referral system Establishment (CARE). This model integrated experiential lessons from intervention strategies tested in previous pilot [16], and strengthens the existing PEC workforce by embedding training and support throughout the healthcare system. It also focuses on providing community-based eye health education and eye disease screening to patients, and establishing a three-tiered eye care referral system at the county, township and village levels. These approaches achieve comprehensive integration across the supply and demand sides, encompassing the full continuum of services from promotion, prevention, diagnosis, treatment, to referral services.

Unlike previous programs, the *Eye CARE Model* leveraged the current policy momentum for establishing County-wide Tight Medical Alliances (CTMAs) in rural China [17,18]. These CTMAs, led by county-level hospitals and supported by lower-tier healthcare institutions, aims to optimize the allocation of county healthcare resources, restructure service processes, and enhance the healthcare system to create a three-tiered linked and synergistically developed model [19,20]. As a public-funded vertical integration model led by local governments, one of primary goals of CTMAs is to strengthen PHC system and deliver universal health coverage (UHC) [19,20]. This organizational structure enhances the linkage and collaboration between clinical and public healthcare services, incentivizes healthcare facilities to prioritize disease prevention and health management, and promotes the rational allocation of resources. By establishing a hierarchical eye care system within the county, CTMAs present a promising pathway to effectively integrate PEC into PHC.

Our study presents the results of both process and outcome evaluations of this multicomponent intervention, which integrates PEC into PHC within the context of CTMAs in rural China, to provide further information for researchers, practitioners, and policymakers on effectiveness and contextual influences on its implementation. First, we applied the Medical Research Council (MRC) process evaluation framework [21] for complex interventions to systematically evaluate the implementation outcomes, and related facilitators and barriers in the healthcare system. Second, we employed a quasi-experimental design using the difference-in-differences (DID) method to assess the impact of the *Eye CARE Model* on eye care service utilization, supplemented by event study methodology to illustrate dynamic effects. To the best of our knowledge, this is the first study that comprehensively evaluates both the process and outcome of integrating PEC into PHC interventions while considering the influence of contextual factors. This research may help guide future efforts to refine and scale up the *Eye CARE Model*, ensuring its effectiveness and sustainability in diverse healthcare settings.

## Methods

### Project overview

#### Background and Study setting

PHC strengthening was considered the key strategy to achieving UHC in the Healthy China 2023 Plan [22]. Enhancing the capacities of rural PHC providers was also documented in the Chinese central authorities issued in the 14^th^ five-year plan period [23]. CTMAs were therefore proposed to address the fragmentation between PHC services (provided by township health centres and villages clinics) and county hospitals services, through changes of leadership commitment, organizational merger and payment mechanism (See Figure S1 in Appendix for a detailed description of the operation of CTMAs). Since 2020, CTMAs pilots were rolled out nationwide with clearly specified guidelines and systematic monitoring of progress and effectiveness [24]. While the policies related to CTMAs are largely similar, the implementation of CTMAs exhibit diverse practices. China’s CTMA pilots provide a unique opportunity to promote PEC in rural areas. Currently, in most PHC facilities, there are no specialized ophthalmologists, nor are there established departments of ophthalmology, which in turn impacts the access and quality of eye care services in these underserved rural areas.

**Figure 1:**
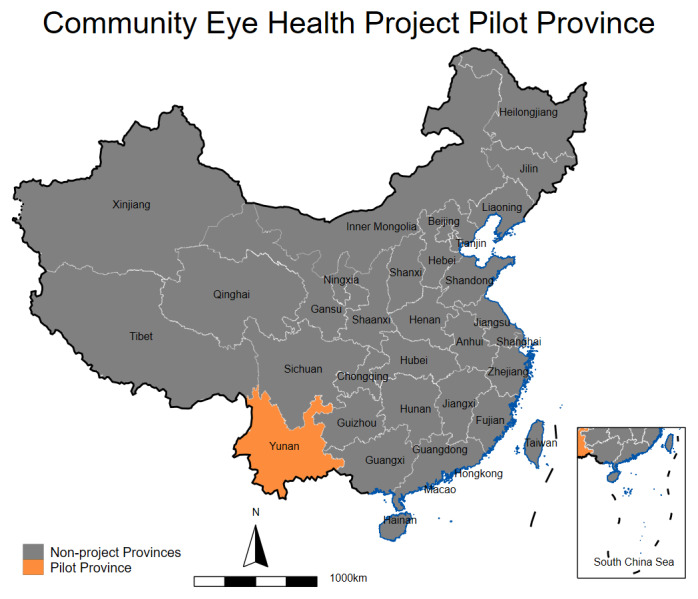
Study area.

We designed a multicomponent intervention for integrating PEC into PHC, the *Eye CARE Model*, leveraging the establishment of CTMAs in rural China. This present paper reports the pilot phase of our project which was conducted at three pilot counties in Yunnan Province between 2021 and 2023 (Figure 1). Yunnan is inhabited by ethnic minorities with a per capita disposable income of approximately $3,718, ranking 28th among 31 provinces [25]. A recent nationally-representative eye disease survey shows that Yunnan has one of the highest severe vision impairment or blindness prevalence rates, yet the lowest rate of eye care utilization—for example, the cataract surgical coverage in Yunnan is only two third of national average (43.4% vs. 62.7%) [4].

In this present study, we applied a Hybrid II effectiveness-implementation quasi-experimental design with mixed-method approach (see details in the following section) [28]. Table 1 provides the basic geographical, demographic, and economic information of our study sites. All three counties are located in mountainous regions with varying proportions of ethnic minority populations. Prior to the implementation of our project, PHC facilities (township health centers and village clinics) in these counties did not provide PEC services due to the lack of trained primary health workers trained and basic equipment. Similar to other regions, the implementation of CTMAs in Yunnan varies significantly in practice across counties in terms governance, organizational integration etc. This context grants the opportunity to (a) evaluates both the process and outcome of integrating PEC into PHC interventions and (b) examine the influence of contextual factors across different specific context. The results of our pilot phase will be used to adapt and refine the design for a multi-center randomized controlled trial (RCT) to test the effectiveness and cost-effectiveness of our PEC model.

**Table 1:** Geography, demography and economic information by pilot counties.


	X COUNTY	Y COUNTY	L COUNTY

Administrative Area (km^2^)	2,425	3,659	1,990

Percentage of Mountainous region (%)	75.5	71.8	61.2

Number of Townships	11	12	9

Population (in tens of thousands)	48.3	44.2	70.2

Male/Female ratio	1.03:1	1.11:1	1.07:1

Percentage of ethnic minorities (%)	18.67	48.48	2.50

GDP (in ten thousand RMB)	2,062,991	1,620,612	3,072,178

Per capita income of urban households (RMB)	42,811	34,137	38,424

Per capita income of rural households (RMB)	15,533	14,984	19,445


Data source: *China County Statistical Yearbook 2022*, https://www.stats.gov.cn/.

#### Intervention components

Like other LMICs, rural areas in China face several significant challenges hindering the integration of PEC into PHC, including: (a) low PEC skill levels, inadequate supervision, insufficient equipment, and a lack of consensus or guidelines on PEC, which restrict the ability of primary care providers [29,30,31]; (b) significant gaps in eye health awareness and literacy, particularly in rural areas, which further contribute to the underutilization of PEC [8,32]; and (c) the absence of an integrated referral system for eye care and the low policy priority of eye health, which limit the resources and capacity of PHC in providing eye care [7,33].

The multicomponent intervention named the *Eye CARE model*, encompasses Capacity building, Awareness raising, and Referral system Establishment (CARE) within the context of CTMAs in rural China. CTMAs provided a governance structure that facilitated coordination, resource allocation, and support for the intervention activities.

- *Capacity building*: A cascade-down Eye health training activities were conducted for township doctors and village doctors through CTMAs. Basic ophthalmic equipment such as slit lamps, flashlights, and vision charts were distributed to PHC facilities. A PEC-focused training was provided on the diagnosis of common eyes conditions, and skills for managing patients with minor eye conditions as well as referral procedures for more complex cases.
- *Awareness raising*: PHC doctors and health workers were coordinated by CTMAs to conduct community-based eye health education and eye disease screenings, aiming to enhance residents’ eye health literacy. This included integrating eye health education into routine public health activities at community level, thereby leveraging the existing health infrastructure and resources managed by CTMAs to maximize outreach and impact.
- *Referral system Establishment*: Leveraging the resources and information system managed by CTMAs, bi-directional county-township-village referral pathways for eye care services were established. Village health clinics were responsible for screening and referring patients, township health centers for diagnosing and treating common basic eye diseases (e.g., conjunctivitis, keratitis) and referring more complex cases, and county-level hospitals for providing further treatment as needed. The integration within CTMAs facilitated the centralized management of referrals, ensuring a seamless and coordinated patient journey across different levels of care.

### Analytical framework

**Figure 2:**
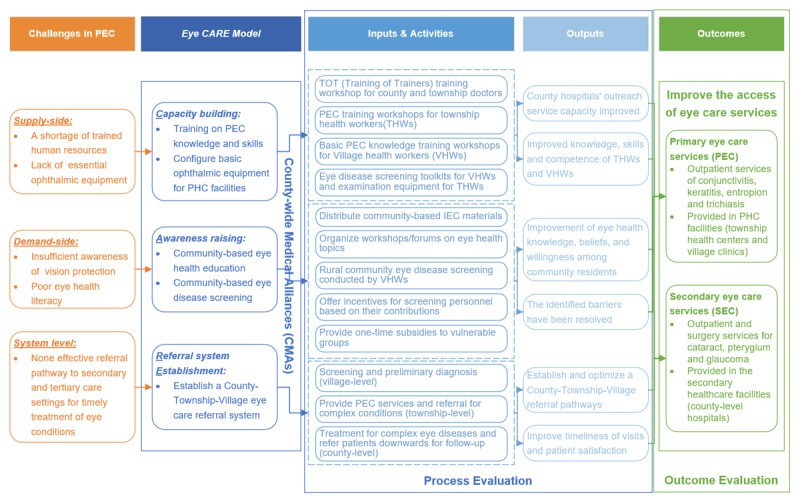
Theory of change and scope of process and outcome evaluation for *Eye CARE* Model in rural China. Figure to illustrate theory of change.

Following the MRC complex intervention evaluation framework [34], we developed our theory of change to guide the process and outcome evaluation of *Eye CARE Model* (Figure 2).

Our process evaluation investigated the interaction between context, intervention components, and implementation indicators [21,35]. We examined the following implementation outcomes: (a) Fidelity, defined as the extent to which the interventions of the *Eye CARE Model* were implemented according to the original protocol including adherence to guidelines, procedures, and intended activities specified in project documents and stakeholder agreements; (b) Reach, referring to the extent to which the target population came into contact with the intervention. It measures how well the intervention is distributed among the intended audience and identifies any disparities in access or engagement; and (c) Adaptation, which assesses the modifications made to the interventions during implementation. This process can be intentional or reactive to unforeseen challenges or opportunities encountered in the field. Documenting adaptations is crucial for evaluating their impact on program outcomes and for informing future scalability and sustainability efforts. Additionally, we studied the contextual factors revolved around the health system’s that could influence the implementation outcomes.

The outcome evaluation was designed as a quasi-experimental study in which the control group includes townships in each pilot county where the *Eye Care Model* has not been introduced. Each county selected approximately half of its townships to implement the project.

### Data collection and analysis

Mixed methods were used to data collection and analysis. We combined a quasi-experimental outcome evaluation with the MRC process evaluation framework of complex intervention [26]. Table S1 in the appendix showed the analysis framework and data collection sources. The study has received ethical approval from Peking University Institutional Review Board Office (IRB 00001052-22097).

#### Process evaluation

In this study, individuals with lived experience were actively involved in the qualitative data collection (Table S2 in Appendix). Community members, including 66 middle school students and 48 individuals aged 50 or above from rural areas, participated in 15 focus group discussions. Their insights were crucial in understanding the local barriers to accessing eye care and the real-world challenges faced by rural populations. Additionally, primary care practitioners and healthcare providers who directly engaged with the intervention contributed valuable perspectives on the implementation and feasibility.

We collected both project documents and qualitative data, focusing on key dimensions of project implementation and exploring variations, facilitators, and barriers. Data analysis combined inductive and deductive approaches, with themes emerging from the theory of change, analytical framework and empirical data, all of which were tested against other data sources to ensure validity. Interviews were coded using NVivo 10.0 software based on a priori themes aligned with our research questions. As the analysis progressed, further themes were added based on emerging insights, and coding was stratified by facility and respondent. Saturation in the qualitative data was determined when no new themes or concepts emerged during interviews, assessed through ongoing review and discussions among the research team. Interviews continued until additional data did not yield new insights. This iterative process ensured the robustness and completeness of the data. All qualitative interview transcripts were translated from Chinese to English (see details in Table S4 in the appendix).

#### Outcome evaluation

The effectiveness of the *Eye CARE Model* on the utilization of eye care services was assessed using a difference-in-differences (DID) approach with two-way fixed effects. The DID method is a robust quasi-experimental design increasingly used in population health intervention evaluations, particularly when randomization is not feasible [36,37]. This approach is especially relevant for complex health system interventions like integrating PEC into general health systems, where real-world conditions are more informative for policymakers than controlled experimental settings.

Our outcome evaluation utilized administrative data from 2019 to 2023, provided by the management information systems of the CTMAs in the three pilot counties, and supplemented by data from the China County Statistical Yearbook (2019–2023) [25]. The intervention group consisted of 18 townships, while the control group comprised 13 townships. Data analysis leveraged the longitudinal nature of the dataset, allowing for the comparison of changes over time between the two groups. The following regression model was used to estimate the causal impact of the intervention:


\[
{{y}_{it}}\ =\ \alpha \ +\ {{\beta}_{1}}Trea{{t}_{it}}\ +\ {{X}_{it}}\ +\ {{\gamma}_{i}}\ +\ {{\delta}_{t}}\ +\ {{\varepsilon}_{it}}
\]

where *y*_it_ is the utilization of eye care services per 10000 people of township i in year t, including primary and secondary eye care services. Primary eye care services include the outpatient services (diagnosis and treatment) of conjunctivitis, keratitis, entropion and trichiasis, provided in PHC facilities (township health centers and village clinics). Secondary eye care services include the outpatient and surgery services for cataract, pterygium and glaucoma, provided in the secondary healthcare facilities (county-level hospitals). *Treat*_it_ is a dummy variable indicating whether the project has been introduced in township i in year t. Under this assumption, the coefficient β_1_ on *Treat*_it_ captures the causal effect of introducing project on the study outcomes. ***X***_it_ is a set of township characteristics including residential population, number of industrial enterprises, and number of stores or supermarkets with a business area of more than 50 m^2^. We cluster the standard errors at the township level. All analyses were conducted in Stata 17. For additional methodological details regarding the inclusion and exclusion criteria, statistical power calculation, and data sources, refer to the Appendix.

## Results

### Process evaluation

#### Fidelity

Based on the detailed data provided in Table 2, the *Eye CARE Model* project has achieved progress beyond expectations in its planned activities. First, the project, under the unified organization of CTMAs, conducted cascade-down training, including training-of-trainers for ophthalmologists at county hospitals and PEC training for township doctors and village doctors. The number of PHWs trained in the three pilot counties exceeded the target by 1.8 to 3.4 times, indicating a significant commitment to capacity building among PHWs, which is also a crucial guarantee for service delivery.

**Table 2:** Progress towards targets (Fidelity & Reach).


	COUNTY X			COUNTY Y			COUNTY L		

***Fidelity***									

	Planned	Implemented	%	Planned	Implemented	%	Planned	Implemented	%

Human resource development	210	355	169%	174	277	159%	166	353	213%

Human resource development-PHW trained	72	242	336%	72	154	214%	72	132	183%

Infrastructure and equipment	2	6	300%	2	6	300%	2	6	300%

Community screening	58750	93734	160%	54750	103939	190%	50750	79765	157%

Eye health education	14000	28985	207%	14000	14297	102%	14000	22785	163%

Establishment of local referral pathway	1	1	100%	1	1	100%	1	1	100%

	Treatment	Control	diff	Treatment	Control	diff	Treatment	Control	diff

The proportion of ophthalmology/ENT departments established in township health centers	4/5	1/5	60%	4/7	0/5	57%	1/5*	0/4	20%

***Reach***									

	population	Population covered	%	population	Population covered	%	population	Population covered	%

Community screening	312760	93734	30%	245022	103939	42%	431333	79765	18%

Eye health education	312760	28985	9%	245022	14297	6%	431333	22785	5%


Source: The *Eye CARE Model* project documents (2021–2023).

The provision of PEC toolkits and ophthalmic equipment has yielded satisfactory results. With external funding, the medical communities in the pilot counties equipped all village health rooms in the pilot townships with basic vision screening tools such as eye charts and flashlights. Additionally, each pilot county provided six township health centers with either desktop or handheld slit lamps. This ensured that grassroots health institutions within the service scope of the medical communities were adequately equipped to conduct eye examinations. Notably, with the project’s assistance, nine out of the seventeen pilot township health centers established independent ophthalmology departments.

Second, community-based eye health education activities and screenings demonstrated ambitious outreach efforts. Over the project cycle, more than 250,000 rural community members underwent eye disease screenings, and over 60,000 individuals participated in various types of eye health education activities organized by CTMAs. However, our qualitative interviews revealed a significant “know-do gap” among rural community residents, especially the elderly. Despite increased knowledge and awareness of eye health, underinvestment in preventive health services such as PEC remains a challenge.

“Even though we now know about the importance of regular eye check-ups and how to take care of our eyes, many of us still don’t go to the hospital until we can barely see. It’s hard to change old habits and make time for something that doesn’t seem urgent.” — Elderly Resident 1, X County

Third, the eye care referral system has been fundamentally established, with all pilot counties successfully creating referral pathways from village clinics to township health centers and then to county hospital ophthalmology departments. The referral methods vary depending on local resource availability. But qualitative interviews with key management personnel of CTMAs indicated the necessity to continue enhancing digital infrastructure to ensure seamless and efficient referral processes across different regions.

“We have established a dedicated ‘Bidirectional Referral Office’ on the ground floor of the outpatient building of the county hospital, where patient appointment times are scientifically allocated through a smart backend system. Additionally, there are designated staff members to receive patients, eliminating the need for registration and simplifying the patient treatment process. Patients only need to bring the referral slip exported from the electronic system directly for medical treatment. Many times, even before our cataract patients arrive, our doctors and nurses are already prepared in the operating room.” — Ophthalmology Department Director 1, Y County

“While we have a basic system in place for referring patients, there are still many issues with connectivity and data sharing. Many doctors here are more accustomed to using phone calls or WeChat to contact the ophthalmology departments at higher-level hospitals for patient referrals. Improving our digital infrastructure would really help streamline these processes and ensure patients get the treatment they need without unnecessary delays.” — Township Health Center Doctor 1, L County

#### Reach

Reach measures the extent of coverage of PEC services across the target population. By July 2023, based on available data at the time of process evaluation, the project had successfully integrated eye care into primary healthcare in 56.3% of townships and 61.1% of villages in the project counties, covering nearly one million rural residents. However, the level of reach varied across the counties due to differing levels of integration within their CTMAs. In X County and Y County, the high integration level of CTMAs allowed for comprehensive community-based eye disease screening, covering over 40% of the rural population. L County’s less integrated CTMA structure resulted in limited coverage, with only 18.5% of the target population undergoing eye disease screening.

#### Adaptability

The three pilot counties strategically adapted the implementation strategies of the comprehensive intervention model based on their unique context of health system and practical challenges. These adaptations included techniques for integration and promotion, service decentralization, and supervision, which enhanced the accessibility and quality of eye care services.

“In Y County, we’ve leveraged innovative technologies to integrate eye care services into our primary healthcare system. Given the transportation challenges in our remote mountainous areas, we also implemented outreach cataract surgeries to ensure no one is left behind.” — Project Manager 1

“Our ophthalmology team at L County hospital regularly conducts teaching and supervision sessions. These efforts have been crucial in elevating the quality of primary eye care services across the region.” — Ophthalmology Department Director 2, L County

Interestingly, all three counties independently integrated regular eye disease screening services into the National Basic Public Health Service Program, facilitating the sustainable provision of primary eye health services. The intervention strategies emphasized building partnerships with local healthcare providers and aligning with the national policy of equalizing basic public health services, thereby integrating eye health services into the primary healthcare system. Under strong policy support, all children under six and adults over 65 can receive vision screenings at primary healthcare institutions. This collaboration has been instrumental in utilizing existing healthcare infrastructure to provide eye health services, enhancing the health system’s capacity to meet the growing demand for eye health services.

The project management team demonstrated adaptability and flexibility in responding to changing circumstances and emerging needs. The project operated an effective and highly participatory responsive management mechanism. The Project Steering Committee reviewed the implementation status half-yearly and made key decisions accordingly.

“The COVID-19 pandemic posed significant challenges, especially for our screening activities. The Project Steering Committee’s dynamic adjustments to project initiation, resource allocation, and goals based on each county’s circumstances were crucial in keeping the project on track.” — Director of the CTMA, X County

#### Facilitators and barriers

The implementation of the *Eye CARE Model* in rural China benefited from several key facilitators across different domains of the health system (Table S5 in the appendix). Strong support from local government and CMAs provided essential political and organizational backing for integrating eye care services into PHC. Significant financial investments from external donors were crucial, offering the necessary seed capital to support various activities, including training, community awareness, and the establishment of referral systems. The CMAs played a vital role in managing and strategically deploying health human resources, facilitating capacity building through training programs for township and village doctors and mentorship from county-level hospital ophthalmologists. Additionally, primary health care facilities were equipped with essential ophthalmic equipment such as slit lamps, flashlights, and vision charts, enhancing their capacity to perform basic eye examinations and manage minor eye conditions. The establishment and strengthening of bidirectional referral systems enabled effective patient referrals from village clinics to township health centers and county hospitals, leveraging available technology. Furthermore, the successful integration of PEC delivery into PHC, supported by the unified efforts of CMAs, included community-based eye health education and disease screening activities, increasing awareness and early detection of eye conditions among rural populations.

Despite these facilitators, several barriers hindered the full implementation (Table S5 in the appendix). Competition for policy priority with other high-profile non-communicable diseases (NCDs) such as diabetes and hypertension limited resources and attention for eye health initiatives. Uncertainty about the long-term financial sustainability of the project once external funding decreases or ends posed a risk to the continuity of primary eye care (PEC) services. High turnover rates among primary health workers created instability in service delivery, necessitating continuous recruitment and training efforts. Limited options for eye care medications at the PHC level often required patients to seek medications from private pharmacies, leading to the potential for unregulated and inappropriate treatments. Poor information technology infrastructure in remote rural areas led to reliance on paper-based referral systems and limited digital records, hindering efficient and accurate patient management. Additionally, varying levels of integration within CMAs resulted in differences in the coverage and effectiveness of eye care services across regions, with some areas achieving comprehensive screening and others lagging behind. The interface between different levels of care could also be strengthened to enhance communication and feedback between primary and other levels of care.

### Outcome evaluation

**Figure 3:**
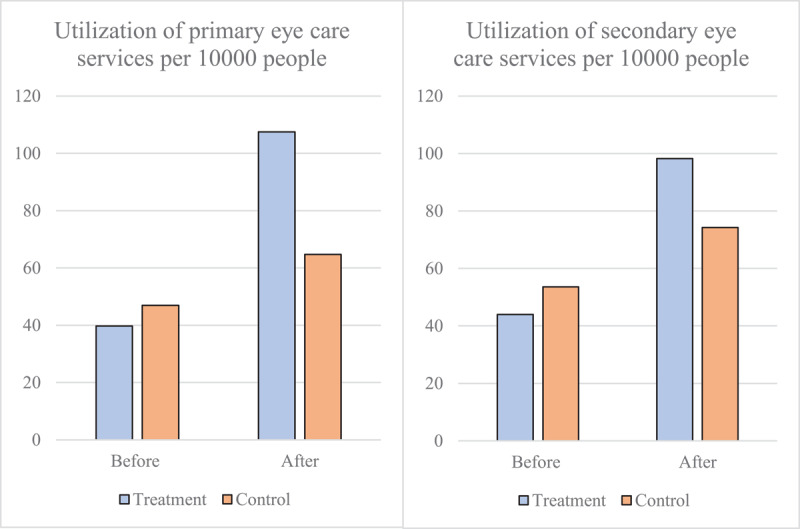
Utilization of eye care services per 10000 people in the treatment and control group.

Table 3 showed the changes in utilization of various eye health services per 10,000 people before and after the project implementation between the intervention and control groups. Prior to the project implementation, the utilization of various eye care services in the intervention group townships was slightly lower than that in the control group townships, but the difference was not significant. Specifically, the utilization of PEC services per 10,000 people was 39.7 visits in the intervention group (compared to 46.9 visits in the control group), and the utilization of secondary eye care services was 44.0 visits (compared to 53.6 visits in the control group). After the project implementation, the utilization of both primary and secondary eye care services in the intervention townships significantly increased compared to the control group (Figure 3). Specifically, primary eye care services increased to 107.5 visits per 10,000 people in the intervention group, which represents a 170.8% increase compared to the baseline. Secondary eye care services increased to 98.2 visits per 10,000 people in the intervention group, a 123.6% increase compared to the baseline.

**Table 3:** Estimated effect of the project on the utilization of eye care services per 10,000 people in the treatment and control groups.


	BEFORE PROJECT IMPLEMENTATION		AFTER PROJECT IMPLEMENTATION		DIFFERENCE-IN-DIFFERENCES EFFECT [95% CI]	P-VALUE
	
	TREATMENT GROUP (n = 18)	CONTROL GROUP (n = 13)	TREATMENT GROUP (n = 18)	CONTROL GROUP (n = 13)		

***Primary eye care services*** ^a^						

Diagnosis and treatment of conjunctivitis	19.4	18.3	63.7	24.7	52.7 [29.8, 75.5]	<0.001

Diagnosis and treatment of keratitis	16.8	23.5	35.5	31.1	20.6 [9.6, 31.7]	0.001

Diagnosis and treatment of entropion	1.0	1.6	2.2	2.9	0.7 [–0.1, 1.5]	0.094

Diagnosis and treatment of trichiasis	2.5	3.4	6.1	5.9	1.9 [0.4, 3.5]	0.017

Total primary eye care services	39.7	46.9	107.5	64.7	75.9 [44.4, 107.4]	<0.001

***Secondary eye care services*** ^b^						

Cataract surgery	7.3	8.4	14.2	10.4	6.7 [4.4, 8.9]	<0.001

Cataract outpatient	15.2	15.5	36.1	21.9	22.3 [10.6, 34.0]	0.001

Pterygium surgery	4.5	5.2	8.8	7.3	4.0 [1.8, 6.2]	0.001

Pterygium outpatient	15.0	20.6	33.7	29.6	16.5 [4.5, 28.5]	0.009

Glaucoma outpatient	2.0	3.9	5.3	5.0	2.5 [1.0, 4.0]	0.002

Total surgery services	11.8	13.5	23.0	17.7	10.6 [6.6, 14.7]	<0.001

Total outpatient services	32.2	40.1	75.2	56.5	41.3 [18.3, 64.4]	0.001

Total secondary eye care services	44.0	53.6	98.2	74.2	52.0 [25.6, 78.3]	<0.001


Note: a) Primary eye care services include the outpatient services (diagnosis and treatment) of *conjunctivitis, keratitis, entropion* and *trichiasis*, which can be provided in the primary healthcare facilities (township health centers and village clinics). b) Secondary eye care services include the outpatient and surgery services of *cataract, pterygium* and *glaucoma*, which can be provided in the secondary healthcare facilities (county-level hospitals). c) Data are mean of utilization of eye care services per 10000 people, or as indicated. d) The results of Difference-in-differences effect are based on two-way fixed effects (TWFE) model, with control variables including a set of township characteristics including residential population, number of industrial enterprises, and number of stores or supermarkets with a business area of more than 50 m^2^. We cluster the standard errors at the township level.

**Figure 4:**
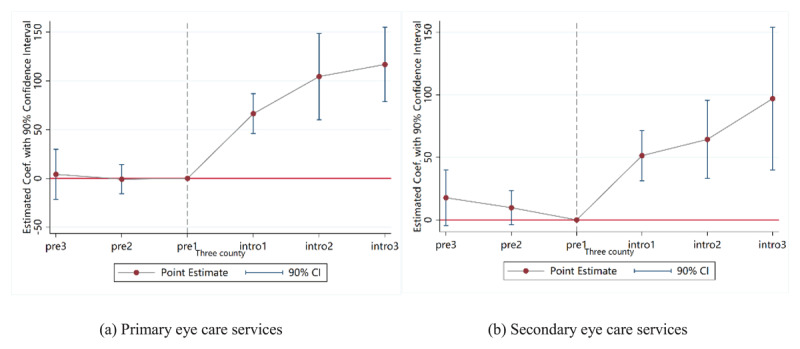
The dynamic effects on the utilization outcomes of eye care services.

Table 3 also reported the regression results of the DID method using a two-way fixed effects model. On average, the *Eye CARE Model* increased the utilization of primary eye care services by approximately 76 visits per 10,000 people and secondary eye care services by approximately 52 visits per 10,000 people. Within secondary eye care, outpatient visits increased by 41 visits and surgeries by 11 visits per 10,000 people. Then we employed the event study method to analyze the dynamic effects (Figure 4), revealing that the impact of the project on increasing eye care service utilization became more significant over time. Figure 4 also showed that the treatment and control groups were not significantly different before the intervention.

Table 4 reported DID regression results on the impact on primary and secondary eye care services (including outpatient and surgical services for various eye conditions) in the three pilot counties. Overall, the *Eye CARE Model* showed statistically significant improvements in the utilization of eye care services across the three pilot counties. Specifically, in X County, the project increased the utilization of PEC services by approximately 110 visits per 10,000 people and secondary eye care services by 108 visits per 10,000 people. In X County, residents in the intervention group showed a significant increase of 82 visits per 10,000 people in PEC service utilization and a significant increase of 28 visits per 10,000 people in secondary eye care service utilization. The corresponding estimates in L County were 3 visits and 10 visits, respectively. Figure S2 in the Appendix also demonstrates the results of dynamic effect analysis, indicating that implementing projects through closely integrated CTMAs can better integrate PEC into PHC, thereby significantly improving the utilization of eye care services compared to loosely organized CTMAs.

**Table 4:** The heterogeneity of the effect on eye care services utilization by county.


	ALL COUNTIES	COUNTY X	COUNTY Y	COUNTY L

***Primary eye care services***				

Diagnosis and treatment of conjunctivitis	52.67*** (4.71)	84.28*** (4.81)	51.55*** (3.20)	1.29* (1.94)

Diagnosis and treatment of keratitis	20.61*** (3.81)	20.44*** (4.38)	28.86** (2.21)	1.51** (2.78)

Diagnosis and treatment of entropion	0.71* (1.73)	1.31* (1.96)	1.17** (2.56)	–0.11 (–0.39)

Diagnosis and treatment of trichiasis	1.92** (2.52)	3.82*** (5.56)	0.80* (1.96)	0.07 (0.17)

Total primary eye care services	75.90*** (4.92)	109.85*** (5.42)	82.37** (3.09)	2.75*** (3.41)

***Secondary eye care services***				

Cataract surgery	6.65*** (5.98)	10.96*** (5.19)	4.24*** (3.85)	3.07** (3.14)

Cataract outpatient	22.31*** (3.89)	47.61*** (4.24)	10.49*** (3.25)	2.77** (3.33)

Pterygium surgery	3.99*** (3.69)	7.28*** (3.84)	3.06*** (6.20)	1.97** (2.92)

Pterygium outpatient	16.48*** (2.81)	37.89*** (9.18)	9.37*** (4.02)	2.90*** (4.01)

Glaucoma outpatient	2.52*** (3.41)	3.82* (2.13)	0.52 (0.54)	–0.29 (–1.03)

Total surgery services	10.64*** (5.37)	18.23*** (5.76)	7.31*** (6.17)	5.04*** (6.13)

Total outpatient services	41.31*** (3.66)	89.32*** (6.12)	20.35*** (5.66)	5.38*** (8.34)

Total secondary eye care services	51.95*** (4.03)	107.55*** (7.15)	27.66*** (7.26)	10.42*** (7.31)

Observations	155	50	60	45

ControlVars	YES	YES	YES	YES

Township FE	YES	YES	YES	YES

Year FE	YES	YES	YES	YES


Notes: Robust t-statistics in parentheses; *** p < 0.01, ** p < 0.05, * p < 0.1.

## Discussion

This study evaluates the *Eye CARE Model*, which integrates PEC into PHC in the context of CTMAs in rural China. The outcome evaluation reveals a significant increase in the utilization of both primary and secondary eye care services in the intervention townships, which is one of the most notable results of this study. These results are highly significant, as they demonstrate a substantial improvement in access to essential eye care services for populations that previously faced significant barriers to care. This is especially important in rural settings where healthcare resources, including specialized services such as eye care, are often scarce. The increased utilization of PEC services addresses a critical gap in the existing healthcare system, contributing to the early detection, diagnosis, and treatment of eye conditions that could otherwise lead to visual impairment or blindness [1,2,3,4]. In rural China, where surgical interventions like cataract surgery are often underutilized, this increase indicates that the *Eye CARE Model* is not only improving access to preventive and basic care but is also facilitating referrals to higher-level facilities for more complex treatments. These outcomes underscore the importance of integrating PEC into the broader health system, which ensures a more seamless continuum of care across different levels of service delivery [1,2,26,27].

The process evaluation results underscore the successful implementation of key components of the model, including capacity building, community awareness campaigns, and the establishment of a referral system, highlighting the progress of key intervention activities, implementation approaches, and an analysis of contextual factors influencing implementation. First, integrating new skills and practices into routine service delivery is inherently complex, necessitating not only capacity building for service providers but also increased community awareness, the establishment of trust, and the provision of essential support systems [13,14,15,38]. Previous PEC strengthening approaches have often underestimated the complexity of context. For instance, a cluster RCT in Nepal demonstrated that despite efforts to train community health volunteers in diagnosing and preventing corneal abrasions, the lack of trust in volunteer service quality and inadequate incentives for service providers, coupled with a failure to fully consider the local context, resulted in no significant improvement in service utilization or eye health outcomes [41]. Similarly, a women’s health worker program in Pakistan, which included basic eye care training for female health workers and aimed to increase screening and community referrals, struggled due to unclear referral pathways and insufficient contextual integration, making referrals to higher-level healthcare facilities challenging [20].

Building on the contextual factors learned from previous (failed) studies, our *Eye CARE Model* applied a holistic triple-intervention solution that addressed health system complexities. By focusing on enhancing the capabilities of PHWs through equipment provision and cascade-down training within the support of CTMAs, we ensure a robust improvement on the access to quality eye care. Moreover, our model emphasizes fostering community engagement and trust through continuous contact and awareness initiatives [5,6,7]. This proactive approach not only increases awareness but also encourages community members to utilize services provided by trained healthcare personnel. Additionally, leveraging the existing CTMAs structure strengthens the referral system, ensuring timely and appropriate care at higher-level healthcare facilities. This integration into the existing healthcare system is crucial for addressing the limitations identified in previous studies and for the sustainable improvement of PEC delivery in rural areas [7,8,9,10].

Another key lesson from the implementation of the *Eye CARE Model* is the importance of leveraging the hierarchical, vertically integrated structure of CTMAs as a platform for complex interventions. While similar studies in India have provided valuable insights into integrating PEC into PHC systems, particularly with robust population data, our study contributes uniquely by utilizing CTMAs to enhance synergies across different healthcare levels, fostering more cohesive service delivery. Both the Indian studies and our work share a focus on community-based interventions and strengthening referral systems. However, the *Eye CARE Model*’s use of CTMAs uniquely addresses the fragmentation often observed between PHC and hospitals in LMICs by promoting vertical integration [39]. The CTMAs addresses this fragmentation through organizational mergers and the promotion of autonomy, aligning previously conflicting interests to improve service delivery. Crucially, local government leadership is vital in CTMA implementation. Effective leadership mobilizes resources, ensures multi-sectoral coordination, and sustains engagement from all stakeholders, from government to local communities. Furthermore, strong leadership is key to securing political support, which is essential for the long-term sustainability of the intervention.

Like any other PHC or even broad health system strengthening strategy, how to sustain the initial implementation success in the long-run is challenging. Given the limited resource in most LMICs settings, eye health has to compete with broader public healthcare needs for prioritization and resources [1,2,40]. The initiation of most PEC strengthening programs in LMICs heavily relies on funding from external donors, lacking sustainability when programs end. It is therefore crucial to identify policy leverage points where eye health can intersect with broader public health issues and to embed eye health within the broader healthcare system strategically. In our case, we integrated community-based eye health screening and education within broader public health priorities in China’s Equalization of Basic Public Health Services (EBPHS) that include routine basic health literacy promotion and physical examinations [41]. In this way, we expanded our program as a stand-alone eye health program to the public health services. Additionally, future sustainability will require a combination of economic incentives, such as screening subsidies, and non-economic incentives, like career development plans. Indeed, as a result of the projected success of this initiative, Xinjiang and Yunnan provinces have launched regional pilot projects for multidimensional PEC strengthening, with the possibility of expansion if successful.

Our study had several strengths, and some limitations. It is one of the first comprehensive evaluations of PEC interventions that combines a quasi-experimental outcome evaluation with the MRC process evaluation framework of complex intervention. Few studies have focused on integrating eye care into general primary healthcare using standardized evaluation frameworks. Although some previous work has utilized the MRC framework, these studies often lacked quantitative evidence on effectiveness from RCTs or quasi-experimental designs related to eye care implementation research. Our approach highlights the dynamic relationship between the intervention and the context in which it is implemented. Despite the non-random allocation of the intervention, we employed a DID quasi-experimental design. This approach relies on weaker assumptions compared to most quasi-experimental methods and enhances the external validity of the results in contrast to RCTs. This strengthens our confidence in the results. Second, our study did not incorporate health outcomes as part of the evaluation. Instead, we measured a broad range of upstream outcomes, including implementation outcomes, which may indicate the intervention’s potential to improve health. This focus on upstream metrics allows for a comprehensive understanding of the intervention’s initial impact, paving the way for future research to explore direct health outcomes. Third, the qualitative interview data may be subject to bias. To mitigate this, we employed triangulation methods aimed at cross-verifying data sources to enhancing data reliability. Furthermore, interviews conducted from the perspectives of multiple stakeholders provided a well-rounded insight into the intervention’s effects.

## Conclusion

The *Eye CARE Model*, rigorously designed and implemented based on existing evidence, has significantly improved the utilization of primary and secondary eye care services in rural China, demonstrating its potential to enhance health outcomes. The seamless integration of multi-component interventions with the existing hierarchical vertically integrated healthcare system reached rural community residents in China to address unmet needs for eye care services, and has achieved adaptive adjustments tailored to the local context and relatively high implementation fidelity. It is necessary to explore the adaption of the *Eye CARE Model* in other settings and for other chronic non-communicable diseases to address the widespread fragmentation of health systems in LMICs.

## Data Accessibility Statement

The datasets and materials generated and analyzed during the current study are available from the corresponding author on reasonable request and subjected to the approval from the ethics review committee.

## Additional File

The additional file for this article can be found as follows:

10.5334/ijic.8972.s1 Supplementary File 1. Figures S1 and S2; Tables S1–S5; Inclusion and Exclusion Criteria for Townships.

## Abbreviations

CTMAs, County-wide Tight Medical Alliances

DID, difference-in-differences

EBPHS, Equalization of Basic Public Health Services

LMICs, Low- and Middle-Income Countries

MRC, Medical Research Council

NCDs, Non-Communicable Diseases

PEC, Primary Eye Care

PHC, Primary Healthcare

RCT, Randomized Controlled Trial

VI, Visual Impairment

## References

[1] Burton MJ, Ramke J, Marques AP, Bourne RRA, Congdon N, Jones I, et al. The Lancet Global Health Commission on Global Eye Health: vision beyond 2020. The Lancet Global Health. 2021;9:e489–551. DOI: 10.25259/IHOPEJO_15_2021 33607016 PMC7966694

[2] World report on vision [Internet]. [cited 2024 Apr 29]. Available from: https://www.who.int/publications-detail-redirect/9789241516570

[3] Universal eye health: a global action plan 2014–2019 [Internet]. [cited 2024 Jun 25]. Available from: https://www.who.int/publications/i/item/universal-eye-health-a-global-action-plan-2014-2019

[4] Yasmin S, Schmidt E. Primary eye care: opportunities for health system strengthening and improved access to services. International Health. 2022;14:i37–40. DOI: 10.1093/inthealth/ihab062 35385864 PMC8986357

[5] Keel S, Evans JR, Block S, Bourne R, Calonge M, Cheng C-Y, et al. Strengthening the integration of eye care into the health system: methodology for the development of the WHO package of eye care interventions. BMJ Open Ophthalmology. 2020;5:e000533. DOI: 10.1136/bmjophth-2020-000533 PMC7418692 32821853

[6] Yasmin S, Schmidt E. Primary eye care: opportunities for health system strengthening and improved access to services. International Health. 2022;14:i37–40. DOI: 10.1093/inthealth/ihab062 35385864 PMC8986357

[7] Lee L, Moo E, Angelopoulos T, Dodson S, Yashadhana A. Integrating eye care in low-income and middle-income settings: a scoping review. BMJ Open. 2023;13:e068348. DOI: 10.1136/bmjopen-2022-068348 PMC10230923 37236663

[8] Solomon SD, Shoge RY, Ervin AM, Contreras M, Harewood J, Aguwa UT, et al. Improving Access to Eye Care: A Systematic Review of the Literature. Ophthalmology. 2022;129:e114–26. DOI: 10.1016/j.ophtha.2022.07.012 36058739

[9] Keel S, Lingham G, Misra N, Block S, Bourne R, Calonge M, et al. Toward Universal Eye Health Coverage—Key Outcomes of the World Health Organization Package of Eye Care Interventions: A Systematic Review. JAMA Ophthalmology. 2022;140:1229–38. DOI: 10.1001/jamaophthalmol.2022.4716 36394836

[10] Courtright P, Seneadza A, Mathenge W, Eliah E, Lewallen S. Primary eye care in sub-Saharan African: do we have the evidence needed to scale up training and service delivery? Ann Trop Med Parasitol. 2010;104:361–7. DOI: 10.1179/136485910X12743554760225 20819303

[11] Aghaji A, Burchett H, Hameed S, Webster J, Gilbert C. The Technical Feasibility of Integrating Primary Eye Care Into Primary Health Care Systems in Nigeria: Protocol for a Mixed Methods Cross-Sectional Study. JMIR Res Protoc. 2020;9:e17263. DOI: 10.2196/17263 33107837 PMC7655465

[12] Aragie S, Wittberg DM, Tadesse W, Dagnew A, Hailu D, Chernet A, et al. Water, sanitation, and hygiene for control of trachoma in Ethiopia (WUHA): a two-arm, parallel-group, cluster-randomised trial. The Lancet Global Health. 2022;10:e87–95. DOI: 10.1016/S2214-109X(21)00409-5 34919861 PMC9360557

[13] O’Brien KS, Byanju R, Kandel RP, Poudyal B, Gonzales JA, Porco TC, et al. Village-integrated eye workers for prevention of corneal ulcers in Nepal (VIEW study): a cluster-randomised controlled trial. The Lancet Global Health. 2022;10:e501–9. 35303460 10.1016/S2214-109X(21)00596-9 PMC9814976

[14] Bechange S, Schmidt E, Ruddock A, Khan IK, Gillani M, Roca A, et al. Understanding the role of lady health workers in improving access to eye health services in rural Pakistan – findings from a qualitative study. Archives of Public Health. 2021;79:20. DOI: 10.1186/s13690-021-00541-3 33597017 PMC7890803

[15] Bechange S, Buttan S. Effectiveness of community-based eye care: process and considerations. The Lancet Global Health. 2022;10:e451–2. DOI: 10.1016/S2214-109X(22)00032-8 35303442

[16] Dong X, Zhao Z, Ma X. Evaluation Report of Promoting Eye Health Through Community Participation in Rural Yunnan, China. Fred Hollows Foundation; 2024.

[17] China’s National Health Commission. Notice on Promoting the Construction of County-wide Tight Medical Alliances [Internet]. [cited 2024 Apr 29]. Available from: https://www.gov.cn/zhengce/zhengceku/2019-10/08/content_5437020.htm

[18] China’s National Health Commission. Guiding Opinions on Comprehensively Promoting the Construction of County-wide Tight Medical Alliances [Internet]. [cited 2024 Apr 29]. Available from: https://www.gov.cn/zhengce/zhengceku/202312/content_6923447.htm

[19] Zhang M, Du X, Jia G, Xia Q, Xu Y, Wu J, et al. Comparative Study on the Satisfaction of Healthcare Service Providers with the Synergistic Development of Rural Healthcare Systems in China: Medical Alliance Counties vs. Non-Medical Alliance Counties. Int J Integr Care. 24:26. DOI: 10.5334/ijic.7677 38911946 PMC11192093

[20] Ran Y, Gao H, Han D, Hou G, Chen Y, Zhang Y. Comparison of inpatient distribution amongst different medical alliances in a county: a longitudinal study on a healthcare reform in rural China. Int J Equity Health. 2020;19:142. DOI: 10.1186/s12939-020-01260-x 32819362 PMC7441726

[21] Moore GF, Audrey S, Barker M, Bond L, Bonell C, Hardeman W, et al. Process evaluation of complex interventions: Medical Research Council guidance. BMJ. 2015;350:h1258. DOI: 10.1136/bmj.h1258 25791983 PMC4366184

[22] The State Council of China. Outline of the ‘Healthy China 2030’ Plan [Internet]. [cited 2024 Jul 31]. Available from: https://www.gov.cn/zhengce/2016-10/25/content_5124174.htm

[23] The State Council of China. 14th Five Year Plan for National Health [Internet]. [cited 2024 Jul 31]. Available from: https://www.gov.cn/zhengce/content/2022-05/20/content_5691424.htm

[24] The State Council of China. Notice on Issuing the Evaluation Criteria and Monitoring Indicator System for the Construction of County-wide Tight Medical Alliances (Trial) [Internet]. [cited 2024 Jul 31]. Available from: https://www.gov.cn/zhengce/zhengceku/2020-09/18/content_5544471.htm

[25] National Bureau of Statistics. China Statistical Yearbook [Internet]. [cited 2024 Jun 14]. Available from: https://www.stats.gov.cn/sj/ndsj/

[26] Zhao J, Ellwein LB, Cui H, Ge J, Guan H, Lv J, et al. Prevalence of Vision Impairment in Older Adults in Rural China: The China Nine-Province Survey. Ophthalmology. 2010;117:409–416.e1. DOI: 10.1016/j.ophtha.2009.11.023 20079923 PMC6029941

[27] Zhao J, Xu X, Ellwein LB, Cai N, Guan H, He M, et al. Prevalence of Vision Impairment in Older Adults in Rural China in 2014 and Comparisons With the 2006 China Nine-Province Survey. American Journal of Ophthalmology. 2018;185:81–93. DOI: 10.1016/j.ajo.2017.10.016 29102607 PMC6029940

[28] Curran GM, Bauer M, Mittman B, Pyne JM, Stetler C. Effectiveness-implementation Hybrid Designs: Combining Elements of Clinical Effectiveness and Implementation Research to Enhance Public Health Impact. Medical Care. 2012;50:217–26. DOI: 10.1097/MLR.0b013e3182408812 22310560 PMC3731143

[29] Kalua K, Gichangi M, Barassa E, Eliah E, Lewallen S, Courtright P. Skills of general health workers in primary eye care in Kenya, Malawi and Tanzania. Hum Resour Health. 2014;12 Suppl 1:S2. DOI: 10.1186/1478-4491-12-S1-S2 25860909 PMC4108885

[30] Byamukama E, Courtright P. Knowledge, skills, and productivity in primary eye care among health workers in Tanzania: need for reassessment of expectations? Int Health. 2010;2:247–52. DOI: 10.1016/j.inhe.2010.07.008 24037865

[31] Mafwiri MM, Jolley E, Hunter J, Gilbert CE, Schmidt E. Mixed methods evaluation of a primary eye care training programme for primary health workers in Morogoro Tanzania. BMC Nursing. 2016;15:41. DOI: 10.1186/s12912-016-0163-5 27390550 PMC4936119

[32] Capó H, Edmond JC, Alabiad CR, Ross AG, Williams BK, Briceño CA. The Importance of Health Literacy in Addressing Eye Health and Eye Care Disparities. Ophthalmology. 2022;129:e137–45. DOI: 10.1016/j.ophtha.2022.06.034 36058736

[33] Jolley E, Mafwiri M, Hunter J, Schmidt E. Integration of eye health into primary care services in Tanzania: a qualitative investigation of experiences in two districts. BMC Health Serv Res. 2017;17:823. DOI: 10.1186/s12913-017-2787-x 29237503 PMC5729236

[34] Skivington K, Matthews L, Simpson SA, Craig P, Baird J, Blazeby JM, et al. A new framework for developing and evaluating complex interventions: update of Medical Research Council guidance. BMJ. 2021;374:n2061. DOI: 10.1136/bmj.n2061 34593508 PMC8482308

[35] Penn-Kekana L, Powell-Jackson T, Haemmerli M, Dutt V, Lange IL, Mahapatra A, et al. Process evaluation of a social franchising model to improve maternal health: evidence from a multi-methods study in Uttar Pradesh, India. Implementation Sci. 2018;13:124. DOI: 10.1186/s13012-018-0813-y PMC6154932 30249294

[36] Tougher S, Dutt V, Pereira S, Haldar K, Shukla V, Singh K, et al. Effect of a multifaceted social franchising model on quality and coverage of maternal, newborn, and reproductive health-care services in Uttar Pradesh, India: a quasi-experimental study. The Lancet Global Health. 2018;6:e211–21. DOI: 10.1016/S2214-109X(17)30454-0 29275135

[37] van Roozendaal H, Derickx K, Ponnet K, Deforche B, Thienpondt A, Glazemakers I, et al. Process and outcome evaluation of a social norms approach intervention on alcohol use among Flemish university students: a quasi-experimental study. Archives of Public Health. 2024;82:45. DOI: 10.1186/s13690-024-01265-w 38549095 PMC10976709

[38] Rono H, Bastawrous A, Macleod D, Mamboleo R, Bunywera C, Wanjala E, et al. Effectiveness of an mHealth system on access to eye health services in Kenya: a cluster-randomised controlled trial. Lancet Digit Health. 2021;3:e414–24. DOI: 10.1016/S2589-7500(21)00083-2 34167763 PMC8239618

[39] Xu J, Yuan B. China Case Study for The Lancet Global Health Commission on financing primary health care, unpublished. London School of Hyigene & Tropical Medicine; 2022.

[40] Yip JLY, Bright T, Ford S, Mathenge W, Faal H, Dushime T, et al. Process evaluation of a National Primary Eye Care Programme in Rwanda. BMC Health Serv Res. 2018;18:950. DOI: 10.1186/s12913-018-3718-1 30526579 PMC6286556

[41] Yuan B, Balabanova D, Gao J, Tang S, Guo Y. Strengthening public health services to achieve universal health coverage in China. BMJ. 2019;365:l2358. DOI: 10.1136/bmj.l2358 31227480 PMC6598722

