# Supplementary material for: Process and Outcome Evaluation of Integrating Primary Eye Care into Primary Healthcare: A Quasi-Experimental Study in Rural China
Source: Int J Integr Care. 2026 Feb 13;26(1):6. doi: 10.5334/ijic.8972 (PMC12904124; doi:10.5334/ijic.8972)
Supplement: Supplementary File 1. — Figures S1 and S2; Tables S1–S5; Inclusion and Exclusion Criteria for Townships. [file ijic-26-1-8972-s1.pdf]

Process and Outcome Evaluation of Integrating Primary Eye Care into Primary Healthcare: A Quasi-experimental Study in Rural China

Appendix

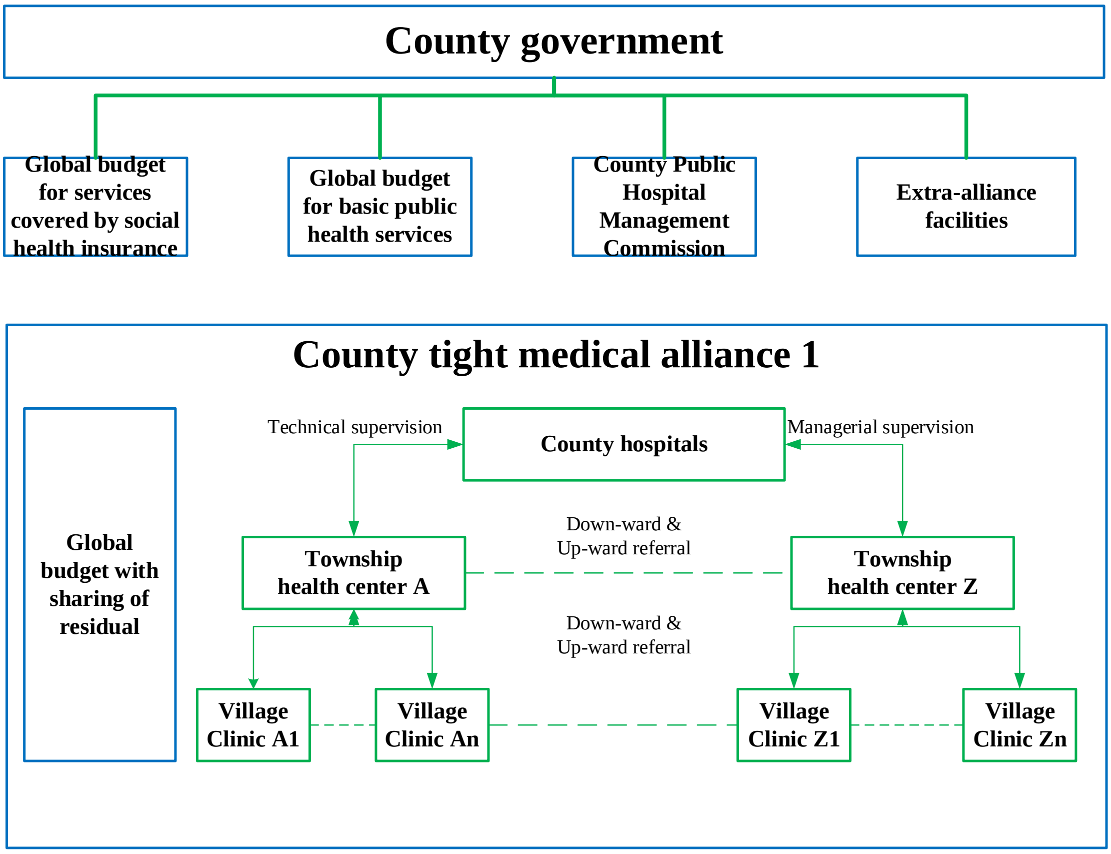

Figure S1. A schematic illustration of the structure of a County Tight Medical Alliance

Figure S2. The dynamic effects on the utilization outcomes of eye care services

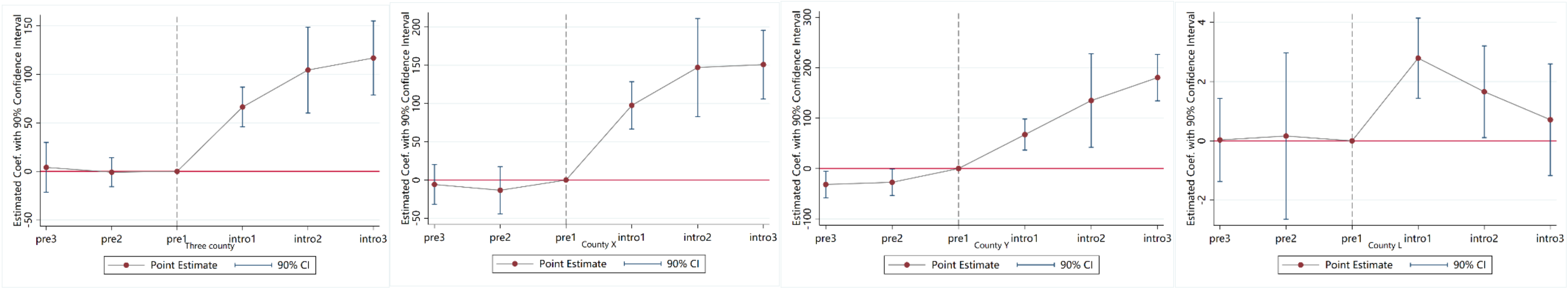

(a) Primary eye care services

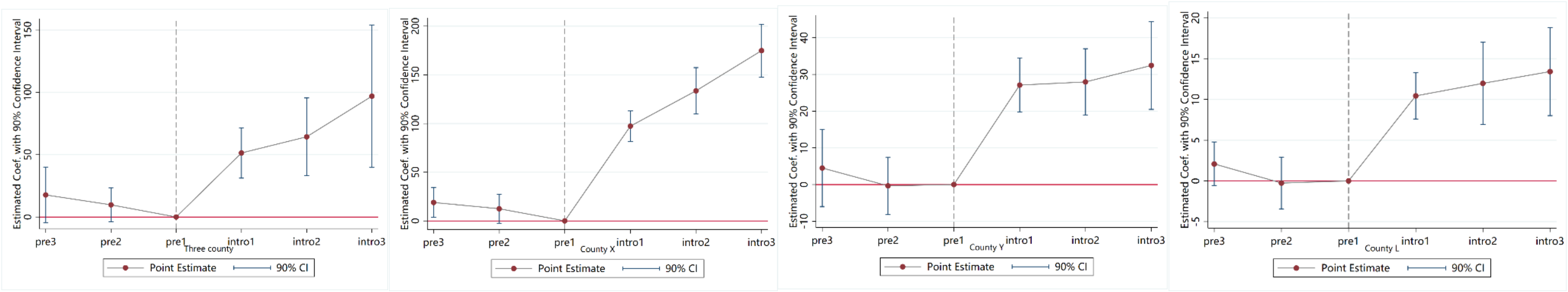

(b) Secondary eye care services

Table S1: Analytical Framework and Data Collection

Domain	Element or indicator for analysis	Data collection
Context		
Healthcare systems	Construction status of County-based Medical Alliance in counties<br>Assessment of eye health service delivery system and capacity (healthcare workforce, information systems, financing, medical products and technologies, etc.)	Stakeholders interviews<br>Documents<br>Literature review
Socioeconomic factors	The level of economic development, resident income level, employment status, distribution of population by age, gender, and ethnicity, etc.	Stakeholders interviews<br>County Statistical Yearbook of China
Geographical factors	Topography and terrain, geographic location and transportation conditions, Climate and weather conditions	Stakeholders interviews<br>County Statistical Yearbook of China
External market environment	Access to eye care services<br>- Non-public healthcare systems (private hospitals, private eyeglass shops)<br>- Non-local (out-of-county) healthcare institutions	Stakeholders interviews<br>Structured observations
Implementation (Process)		
Fidelity	Progress towards targets<br>-Number of training participants<br>-Quantity of equipment/tools distributed<br>-Number of eye health educations<br>-Frequency of regular meetings, etc.	Project documents<br>Routine monitoring data<br>Stakeholders interviews
Dose	The quantity of services delivered, including screening, treatment, referrals	Project documents<br>Routine monitoring data<br>Administrative data from hospitals<br>Stakeholders interviews
Adaptation	Changes to the project	Project documents<br>Stakeholders interviews
Reach	Geographical spread of services<br>The population covered by services	Routine monitoring data<br>Project documents
Outcomes		
Quality	People-centeredness<br>Timeliness<br>Equity<br>Effectiveness	Structured observations<br>Questionnaire surveys<br>Project documents<br>Stakeholders interviews

Access	Utilization of eye care services	Administrative data from hospitals

Table S2: List of Interviewees

In-depth Interviews with Key Stakeholders from the Supply Side

Stakeholder	Personnel coding	Title	Gender
County-level hospital staff	C11	Director of People's Hospital of X County	Male
	C12	Director of the County-based Medical Alliance in X County	Female
	C13	Director of Ophthalmology Department, X County People's Hospital	Female
	C21	Director of the County-based Medical Alliance in Y County	Female
	C22	Director of Ophthalmology Department, Y County People's Hospital	Male
	C23	Director of the Information Department, Y County People's Hospital	Male
	C31	Deputy Director of the First People's Hospital of L County	Male
	C32	Director of Ophthalmology Department, First People's Hospital of L County	Male
	C33	Head Nurse of the Ophthalmology Department, First People's Hospital of L County	Female
Township health center staff	T11	Director of the Township Health Center in X County	Male
	T12	Ophthalmologist of the Y Township Health Center in X County	Male
	T13	Director of the Township Health Center in X County	Male
	T14	Ophthalmologist of the Township Health Center in X County	Male
	T21	Deputy Director of the Township Health Center in Y County	Female
	T22	Ophthalmologist of the Township Health Center in Y County	Male
	T23	Director of the Township Health Center in Y County	Male
	T24	Ophthalmologist of the Township Health Center in Y County	Female
	T25	Director of the Township Health Center in L County	Male
	T26	Director of the Township Health Center in L County	Male
	T27	Deputy Director of the Township Health Center in L County	Male
Village clinic doctors	V11	Village doctor, X County	Female
	V12	Village doctor, X County	Female
	V13	Village doctor, X County	Female
	V21	Village doctor, Y County	Female
	V22	Village doctor, Y County	Male
	V23	Village doctor, Y County	Female
	V24	Village doctor, Y County	Male
	V25	Village doctor, Y County	Male
	V26	Village doctor, Y County	Male
	V31	Village doctor, L County	Male
	V32	Village doctor, L County	Female

	V33	Village doctor, L County	Male

Focus Group Discussion with Key Stakeholders from the Demand Side

Group	Participants	Interview location
1-5	26 middle school students (13 male, 13 female)	Xiangyun County
6	9 rural community members aged 45 and above (4 male, 5 female)	Xiangyun County
7-8	15 rural community members aged 45 and above (7 male, 8 female)	Xiangyun County
9-10	20 middle school students (10 male, 10 female)	Xiangyun County
11	4 rural community members aged 45 and above (1 male, 3 female)	Yun County
12	10 rural community members aged 45 and above (5 male, 5 female)	Yun County
13	10 middle school students (5 male, 5 female)	Yun County
14	10 middle school students (5 male, 5 female)	Luliang County
15	10 rural community members aged 45 and above (5 male, 5 female)	Luliang County

Table S3: Qualitative Interview GUIDE

Qualitative Interview Guide Based on the Theory of Change

Interview Guide with Leaders of County People's Hospital		
Analysis Dimension	Theme	Key Questions
Context	Basic Information	1.1 Understand and record personal characteristics<br>1.2 Basic information about the hospital<br>1.3 Status of ophthalmology construction
	County-based Medical Alliance	2.1 Brief introduction to the development history of the County-based Medical Alliance (record milestone events)<br>2.2 What innovations have been made in local County-based Medical Alliance construction in terms of organization and technology, and what resources have been integrated?<br>2.3 What support has been obtained during the County-based Medical Alliance reform process, and what challenges have been encountered?<br>2.4 Evaluation of the current closeness of the County-based Medical Alliance<br>2.5 The impact of County-based Medical Alliance construction on hospital performance and development<br>2.6 How is the project combined with County-based Medical Alliance construction, and how does it benefit from the County-based Medical Alliance model (such as referral channels)?<br>2.7 The impact of project cooperation on hospital and County-based Medical Alliance construction (capacity improvement, business changes, etc.)<br>2.8 Major issues to be addressed in the current or future County-based Medical Alliance reform and future plans
	COVID-19	3.1 Were the initial stages of the project affected by the COVID-19 pandemic, and how was it resolved?<br>3.2 Did the hospital or County-based Medical Alliance develop and apply digital medical services during the pandemic?
	Market Environment	4.1 Besides hospitals or County-based Medical Alliance units, what other institutions in the area can provide eye health services?<br>4.2 If they exist, are they in a competitive or cooperative relationship, and what are their respective comparative advantages?
	Policy Environment	5.1 What supportive/restrictive policies and regulations are currently in place for community eye health projects?<br>5.2 What work has been done by other relevant units besides the health system?<br>5.3 Eye health-related policies (such as cataract surgery - medical insurance, optometry services, etc.)
Input & Process	Training Activities	6.1 What convenient conditions has the hospital provided for training activities?<br>6.2 The impact of training activities on hospital business capabilities, patient satisfaction, etc.<br>6.3 Any suggestions for the next training activities?
	Referral System	7.1 What is the current referral mode?<br>7.2 Level of grassroots referral rate<br>7.3 Suggestions for optimizing the future referral system

	Health Education	8.1 What health education activities does the hospital currently conduct?<br>8.2 Is eye health education conducted as standalone activities or bundled with related activities?
	Integrated Services	9.1 Besides County-based Medical Alliance members, what other partners are there in the area and what roles do they play?<br>9.2 The implementation of basic public health services in the locality; any integration with project content
Output	Ophthalmic Service Capacity	10.1 Common eye diseases that the hospital can treat<br>10.2 Timeliness of referrals
	Favorable Environment	11.1 Who are the main partners currently, their roles, and potential partners?<br>11.2 Supportive or restrictive policies related to eye health (such as cataract surgery - medical insurance, optometry services, etc.)<br>11.3 Supportive or restrictive policies for County-based Medical Alliance construction (central and local)
Outcome	Service Outcomes	12.1 Patient satisfaction<br>12.2 Changes in the number or proportion of patients seeking medical treatment within the county
	Patient Outcomes	13.1 Occurrence and treatment rates of major local eye diseases

Interview Guide with Eye Department Staff of County People's Hospital		
Analysis Dimension	Theme	Key Questions
Context	Basic Information	1.1 Understand and record personal characteristics<br>1.2 Status of ophthalmology construction
	County-based Medical Alliance	2.1 Cooperation with other units in ophthalmology under the background of the County-based Medical Alliance<br>2.2 How does the project integrate with County-based Medical Alliance construction, and how does it benefit from the County-based Medical Alliance model (such as referral channels)?
	COVID-19	3.1 Were the initial stages of the project affected by the COVID-19 pandemic, and how was it resolved?<br>3.2 Did the hospital or County-based Medical Alliance develop and apply digital medical services during the pandemic, specifically in the ophthalmology department?
	Market Environment	4.1 Besides hospitals or County-based Medical Alliance units, what other institutions in the area can provide eye health services?<br>4.2 If they exist, are they in a competitive or cooperative relationship, and what are their respective comparative advantages?
	Policy Environment	5.1 What supportive/restrictive policies and regulations are currently in place for community eye health projects?<br>5.2 What work has been done by other relevant units besides the health system?<br>5.3 Eye health-related policies (such as cataract surgery - medical insurance, optometry services, etc.)
Input & Process	Training Activities	6.1 What training activities have been organized or participated in, and what are the results?<br>6.2 The impact of training activities on ophthalmic business capabilities, patient satisfaction, etc.

		6.3 Needs or suggestions for the next training activities
	Referral System	7.1 Evaluation of the current referral mode<br>7.2 Level of grassroots referral rate<br>7.3 The role of the county hospital's ophthalmology department in screening activities<br>7.4 Suggestions for optimizing the future referral system
	Health Education	8.1 What eye health education activities does the hospital currently conduct, and how effective are they?<br>8.2 Is eye health education conducted as standalone activities or bundled with related activities?<br>8.3 Current main difficulties and problems
	Integrated Services	9.1 Are there tasks to undertake basic public health services?<br>9.2 Evaluation of the workload in daily work
Output	Ophthalmic Service Capacity	10.1 Qualifications of ophthalmic staff (not limited to ophthalmology)<br>10.2 Scope of ophthalmic services<br>10.3 Status of relevant diagnosis and treatment data<br>10.4 Construction status of the Eye Health Resource Center
	Demand-side Eye Health Literacy and Awareness	11.1 Referral-to-treatment ratio<br>11.2 Changes in overall ophthalmic workload<br>11.3 Assessment of changes in eye health awareness among residents through screening and health education activities
	Favorable Environment	12.1 Supportive or restrictive policies related to eye health (such as cataract surgery - medical insurance, optometry services, etc.)<br>12.2 Favorable policies for ophthalmic construction<br>12.3 Future development plans; desired support
Outcome	Service Outcomes	13.1 Patient satisfaction<br>13.2 Acceptability or attitudes of staff towards the community eye health project model<br>13.3 Evaluation of the convenience and timeliness of referrals or treatments (such as severity of initial treatment for cataract patients, waiting time for surgery, etc.)
	Patient Outcomes	14.1 Screening rates, occurrence rates, and treatment rates of major local eye diseases

Interview Guide with Health Workers in Townships/Villages		
Analysis Dimension	Theme	Key Questions
Context	Basic Information	1.1 Understand and record personal characteristics<br>1.2 Roles in County-based Medical Alliance construction<br>1.3 Main roles in the project
	County-based Medical Alliance	2.1 Division of labor and cooperation under the background of the County-based Medical Alliance<br>2.2 How the project integrates with County-based Medical Alliance construction, and how it benefits from the County-based Medical Alliance model (such as referral channels)?
	COVID-19	3.1 Were the initial stages of the project affected by the COVID-19 pandemic, and how was it resolved?<br>3.2 Did the hospital or County-based Medical Alliance develop and apply

		digital medical services during the pandemic?
Input & Process	Training Activities	4.1 Which training activities have been organized or participated in, and what are the results?<br>4.2 The impact of training activities on ophthalmic business capabilities, etc.<br>4.3 Needs or suggestions for the next training activities
	Referral System	5.1 Evaluation of the current referral mode<br>5.2 Level of grassroots referral rate<br>5.3 The role played in screening activities, and existing difficulties<br>5.4 Any relevant incentives or rewards<br>5.5 Suggestions for optimizing the future referral system
	Health Education	6.1 What eye health education activities are currently conducted, and how effective are they?<br>6.2 Is eye health education conducted as standalone activities or bundled with related activities?<br>6.3 Current main difficulties and problems
	Integrated Services	7.1 Are there tasks to undertake basic public health services?<br>7.2 Evaluation of the workload in daily work
Output	Ophthalmic Service Capacity	8.1 Qualifications of grassroots health workers (not limited to ophthalmology)<br>8.2 Scope of ophthalmic services<br>8.3 Status of relevant diagnosis and treatment data<br>8.4 Construction status of the Eye Health Resource Center
	Demand-side Eye Health Literacy and Awareness	9.1 Referral-to-treatment ratio<br>9.2 Changes in overall ophthalmic workload<br>9.3 Evaluation of changes in eye health awareness among residents through screening and health
	Favorable Environment	10.1 Supportive or restrictive policies related to eye health (such as cataract surgery - medical insurance, optometry services, etc.)<br>10.2 Favorable policies for ophthalmic construction<br>10.3 Future development plans; desired support
Outcome	Service Outcomes	11.1 Patient satisfaction<br>11.2 Acceptability or attitudes of staff towards the community eye health project model<br>11.3 Evaluation of the convenience and timeliness of referrals or treatments
	Patient Outcomes	12.1 Screening rates, occurrence rates, and treatment rates of major local eye diseases

Demand-side Interview Key Questions

For Elderly Residents in the Community:

What eye health services do you know your village clinic/community health service center can provide?

Have you sought or received any eye-related services at the village clinic?

Please rate the eye health services at the village clinic (1-10).

What eye health services do you know your township health center can provide?

Have you sought or received any eye-related services at the township health center?

Please rate the eye health services at the township health center (1-10).

What eye health services do you know your county hospital can provide?

Have you sought or received any eye-related services at the county hospital?

Please rate the eye health services at the county hospital (1-10).

Do you think seeking medical treatment within the county (county-township-village three-level) is convenient? (1-10)
If you have ever sought medical treatment locally due to eye problems, please recall the time from screening to referral and then treatment, and evaluate whether it was timely?
What difficulties have you encountered in the process of seeking medical treatment (e.g., screening-referral-treatment)?
Are you aware of the ongoing community eye health project?
What assistance would you most like to receive in terms of eye health services?
Do you have any suggestions for the community eye health project? For example, the frequency and method of eye examination, the method and content of eye health education activities.

For Local Middle School Students:
What eye health education and promotion activities have been conducted in your school? What impressed you the most?
What aspects of eye care do you want to know about regarding eye care?
Please rate the popularity of eye health promotion and education activities conducted by the school among your classmates (1-10).
If your classmates or friends have eye discomfort, would you actively advise them to see the teacher or community doctor for relevant examinations?

Table S4: Key insider quotes for qualitative interviews

DOMAIN	QUOTES	STAKEHOLDER
Project Management and Coordination	*"We implemented a problem list system and a time-limited supervision system throughout the entire hospital community system, held regular meetings, and provided timely feedback and adjustments to problems that arise in project management and coordination."*	Medical Communities Administrative manager 1, X County
Rural Eye Healthcare System Improvement	*" The training sessions were interactive and practical, which helped us apply the knowledge directly to our work. And the training provided us with valuable insights into identifying common eye conditions and understanding the importance of early intervention."*	Township Doctor 1, Y County
	"Previously, there was a lack of necessary equipment and knowledge, and the treatment of eye diseases was very cautious and conservative. In most cases, I would choose to directly refer upwards. But I feel more confident now in conducting eye screenings and providing basic treatment to patients in my community."	Village Doctor 1, L County
	"We now conduct regular eye screening even in remote villages. This proactive approach has helped in early detection, intervention and prevention."	Village Doctor 2, Y County
	" After the training, we implemented regular eye health awareness campaigns in our villages, which have been well-received by the community. By educating the community about eye hygiene and preventive measures, we've witnessed a positive shift in attitudes towards eye care."	Village Doctor 3, X County
	"Our collaboration with the county-level hospitals has strengthened after the training, allowing for smoother referrals and better coordination of eye care services."	Township Doctor 2, X County
	"We have established a dedicated 'Bidirectional Referral Office' on the	Ophthalmology Director 1, Y County

	ground floor of the outpatient building of the county hospital, where patient appointment times are scientifically allocated through a smart backend system. Additionally, there are designated staff members to receive patients, eliminating the need for registration and simplifying the patient treatment process. Patients only need to bring the referral slip exported from the electronic system directly for medical treatment. Many times, even before our cataract patients arrive, our doctors and nurses are already prepared in the operating room."	
	"Currently, we still find the method of referral via phone or WeChat more convenient and timelier. The referral slip is still in paper form. Due to the existence of two Medical Communities, there are significant obstacles to mutual recognition of referral information."	Township Doctor 3, L County
Follow-up Actions by Rural Residents	*"After the screening, I was referred to a specialist for cataract surgery. Initially hesitant, but realizing the severity of my condition, I decided to undergo the surgery. It has improved my vision significantly."*	Rural Community Elderly 1, X County
	"Although the screening identified my eye condition, the subsequent recommended surgery posed a financial burden."	Rural Community Elderly 2, L County
	"The doctor recommended cataract surgery for me, but my children all work outside the province, and they may not come home for Chinese New Year. There's no one to sign the consent form for me. Moreover, I heard that surgery requires hospitalization, and my bedridden spouse and two four-year-old grandchildren at home need constant care."	Rural Community Elderly 3, X County
	"Our primary healthcare workers have put in a lot of effort. Many elderly cataract patients living in mountainous areas or with mobility issues are personally picked up and dropped off by	Village doctor 4, Y County

	the director of our township health center in his private car."	
**_Rural People's Awareness of Eye Health_**	*"Before the training, we didn't realize the significance of regular eye check-ups. Now, we understand the importance of early detection and treatment."*	Rural Community Elderly 4, L County
	"We learned simple techniques to protect our eyes, like proper handwashing, and knew where to go for specialized care if needed."	Rural Community Elderly 5, X County
	"When the eye health resource center was first set up, only very few people came to the center to participate in eye health education activities and consultations. After communicating with the village committee, our team went to different villages one by one to do the community mobilization and to conduct eye health education work. The numbers of villagers willing to visit the resource center increased dramatically, from less than 10 people a week at the beginning to an average of 30-40 people per week."	Village doctor 5, X County
	"Initially, eye health wasn't a priority in our agenda. However, witnessing the impact of the training on community awareness made us realize its importance in overall public health."	Medical Communities Administrative manager 2, Y County
	"We now prioritize allocating resources for eye health initiatives, the project served as a catalyst for strengthening partnerships and mobilizing resources towards this cause, especially against the backdrop of the upcoming comprehensive implementation of Medical Group construction throughout the county."	Medical Communities Administrative manager 3, L County
**_Eye Health Education at Middle Schools_**	*"I didn't know that too much screen time could harm my eyes. Now, I take breaks and follow the 20-20-20 rule to protect my eyes."*	Student 1, X County
	"After learning about the importance of outdoor play in preventing myopia, I spend more time playing outside, I also remind my friends to take care of their	Student 2, Y County

	eyes."	
	"I shared what I learned with my family, and now we make sure to have regular eye check-ups. I want everyone to know how to take care of their eyes."	Student 3, L County
Equity And Inclusion in The Project	*"I don't believe there are gender differences in eye health medical services. Nowadays, there is hardly any bias favoring males over females, and it seems that in the past two years, there have been even more women coming for cataract surgeries."*	Ophthalmology Director 2, L County

Table S5. Contextual factors for the Eye CARE Model implementation in rural China

**Health system domain**	**Facilitators**	**Challenges**
Leadership and governance	Strong support from local government and County-wide Medical Alliances (CMAs) providing political and organizational backing for the integration of eye care services into primary health care (PHC)	Competition for policy priority with other high-profile non-communicable diseases (NCDs) such as diabetes and hypertension, potentially limiting resources and attention for eye health initiatives
Healthcare financing	Significant financial investments from external donors, which provided the necessary seed capital for the project and supported various activities including training, community awareness, and establishment of referral systems	Uncertainty about the long-term financial sustainability of the project once external funding decreases or ends, posing a risk to the continuity of primary eye care (PEC) services
Health workforce	Effective unified management and strategic deployment of health human resources by CMAs, including capacity building through training programs for township and village doctors, and mentorship from county-level hospital ophthalmologists	High turnover rates among primary health workers, leading to instability in service delivery and necessitating continuous recruitment and training efforts
Medical products and Technologies	Provision of essential ophthalmic equipment, such as slit lamps, flashlights, and vision charts, to primary health care facilities, enhancing their capacity to perform basic eye examinations and manage minor eye conditions	Limited options for eye care medications at the primary health care (PHC) level, often requiring patients to seek medications from private pharmacies, which may lead to unregulated and inappropriate treatments
Information system	Establishment and strengthening of bidirectional referral systems, enabling effective patient referrals from village clinics to township health centers and county hospitals, and leveraging technology where available	Poor information technology infrastructure in remote rural areas, leading to reliance on paper-based referral systems and limited digital records, hindering efficient and accurate patient management
Service delivery	Successful integration of PEC delivery into PHC. Implementation of community-based eye health education and disease screening	Varying levels of integration within CMAs, resulting in differences in coverage and effectiveness of eye care services across regions, with

	activities, including outreach activities such as outreach surgeries in townships and outreach screenings in villages, increasing awareness and early detection of eye conditions among rural populations, supported by the unified efforts of CMAs	some areas achieving comprehensive screening and others lagging behind.<br>Interface between different levels of care could be strengthened – increase communication and feedback between primary and other levels of care

Inclusion and Exclusion Criteria for Townships

The townships included in the study were selected based on specific criteria designed to ensure comparability between intervention and control groups.

Inclusion Criteria:

1. **Geographical Coverage:** Townships located within the three pilot counties (X County, Y County, and L County) in Yunnan Province, China.
2. **Healthcare Infrastructure:** Townships with primary healthcare (PHC) facilities that provide basic healthcare services but did not previously offer comprehensive primary eye care (PEC) services.
3. **Willingness to Participate:** Both intervention and control townships needed to demonstrate a willingness to participate in the study and collaborate with local healthcare authorities for data collection and project implementation.
4. **Population Size:** Townships with a minimum population threshold of 10,000 residents to ensure adequate sample size and variability in the outcome measures.

Exclusion Criteria:

1. **Pre-existing PEC Services:** Townships that already had established primary eye care services were excluded to avoid confounding factors and ensure that any observed effects were due to the intervention rather than pre-existing services.
2. **Inadequate Administrative Data:** Townships with incomplete or inconsistent administrative health data from the past five years (2019-2023) were excluded to ensure accurate analysis of eye care service utilization.
3. **Access Issues:** Townships located in extremely remote areas, where access to healthcare services was severely limited or unreliable, were excluded to prevent data collection challenges and ensure that the intervention could be feasibly implemented.

Selection of Intervention and Control Townships:

- **Intervention Townships:** 18 townships were selected to implement the *Eye CARE Model*, based on their geographic proximity to healthcare centers capable of supporting the intervention, and their alignment with CTMA policy objectives. The intervention group was randomly selected from eligible townships in the pilot counties.
- **Control Townships:** 13 townships were selected as control, with similar population sizes, demographics, and healthcare infrastructure, but without any exposure to the *Eye CARE Model* intervention during the study period. These townships were used to compare pre- and post-intervention data to measure the effect of the *Eye CARE Model.*

Power Calculation

Power calculations were performed to determine the sample size required for detecting significant differences in eye care utilization between the intervention and control groups. The calculation was based on the expected effect size of the intervention, variance in the outcome measures, and the statistical significance level

of 0.05.

Assumptions:

1. **Effect Size:** Based on similar studies in low- and middle-income countries (LMICs) that have used difference-in-differences (DID) methods to evaluate health interventions, we anticipated a moderate effect size of approximately 0.3 standard deviations for changes in primary and secondary eye care utilization.
2. **Significance Level:** A significance level of 0.05 was used for hypothesis testing, consistent with standard practice in health intervention studies.
3. **Power:** A statistical power of 0.80 was selected to ensure an 80% chance of detecting a true effect if it existed.
4. **Intraclass Correlation (ICC):** The intra-township correlation coefficient was assumed to be 0.10 based on previous evaluations of health interventions in similar settings, indicating a moderate degree of correlation within townships.
5. **Attrition Rate:** An attrition rate of 10% was assumed for missing or incomplete data, which is common in longitudinal health intervention studies.

Based on these assumptions, the calculated sample size for each group (intervention and control) was 13 townships, ensuring sufficient power to detect meaningful differences in eye care utilization across the study period. The actual sample included 18 intervention townships and 13 control townships, exceeding the required sample size, which provides additional robustness to the results.

Data Sources

The data for this study were primarily collected from two main sources: administrative data from the County-wide Tight Medical Alliances (CTMAs) and the China County Statistical Yearbook.

1. **Administrative Data from CTMAs (2019-2023):**
   - The CTMAs provided comprehensive data on the utilization of primary and secondary eye care services across the pilot counties. This included:
     - **Primary Eye Care Services:** Data on outpatient visits for conditions such as conjunctivitis, keratitis, entropion, and trichiasis, all provided at township health centers and village clinics.
     - **Secondary Eye Care Services:** Data on more specialized services provided at county-level hospitals, including cataract surgeries, pterygium surgeries, and glaucoma treatments.
   - These data were aggregated at the township level and tracked over the study period (2019-2023) to assess pre- and post-intervention changes in service utilization.
2. **China County Statistical Yearbook (2019-2023):**
   - Socio-economic and demographic characteristics were sourced from the China County Statistical Yearbook. These included:
     - **Township-Level Demographics:** Population size, gender distribution, and ethnic composition.
     - **Economic Indicators:** Data on the number of industrial enterprises,

stores, and supermarkets (with a business area of more than 50 m^2) as proxies for economic activity and development.

- These variables were included in the regression model as covariates to control for potential confounding factors that could influence eye care utilization.
